# Supplementary material for: Chemical Ecology of Streptomyces albidoflavus Strain A10 Associated with Carpenter Ant Camponotus vagus
Source: Microorganisms. 2020 Dec 9;8(12):1948. doi: 10.3390/microorganisms8121948 (PMC7763447; doi:10.3390/microorganisms8121948)
Supplement: Supplementary file 1 [file microorganisms-08-01948-s001.pdf]

Chemical ecology of *Streptomyces albidoflavus* strain A10 associated with carpenter ant *Camponotus vagus*

Supplementary information

Anna A. Baranova[1,2], Alexey A. Chistov[3,2], Anton P. Tyurin[1,2], Igor A. Prokhorenko[1,2], Vladimir A. Korshun[1,2], Mikhail V. Biryukov[4,1], Vera A. Alferova[1 ,2,*], Yuliya V. Zakalyukina[5,*]

[1.]Gause Institute of New Antibiotics, B. Pirogovskaya 11, 119021, Moscow, Russia; alferovava@gmail.com (V.A.A.), ap2rin@gmail.com (A.P.T.), anjabaranowa@list.ru (A.A.B.), prig67@mail.ru (I.A.P.), v-korshun@yandex.ru (V.A.K)

[2.]Shemyakin-Ovchinnikov Institute of Bioorganic Chemistry, Miklukho-Maklaya 16/10, 117997, Moscow, Russia; dobr14@yandex.ru (A.A.C)

[3.]Orekhovich Research Institute of Biomedical Chemistry, Pogodinskaya 10, Moscow 119121, Russia;

[4.]Department of Biology, Lomonosov Moscow State University, Moscow, 119991, Russia; metrim@gmail.com (M.V.B)

[5.]Department of Soil Science, Lomonosov Moscow State University, Moscow, 119991, Russia;

*****[.]Correspondence: juline@soil.msu.ru (Y.V.Z.), alferovava@gmail.com (V.A.A.); Tel.: +79175548004 (Y.V.Z), +79266113649 (V.A.A.)

Contents

Figure S1. Utilization of substrates by the paper disk method 3

Figure S2. Phenotypic properties: utilization as sole carbon source (1.0 %, w/v), growth at NaCl (0.6, 1, 5, 8%) and hydrolysis of urea. 4

Figure S3. Light microscopy photo of Rectiflexibiles spore chains of strain A10 after cultivation for 2 weeks at 28ºC on ISP3 medium (magnification 400)............ 6

Figure S4. Antagonistic activity of the *Streptomyces* sp. A10 using dual-culture assay 7

Figure S5. Antagonistic activity of the *Streptomyces* sp. strain A10 using the cross streak method 8

Figure S6. HPLC chromatogram of purified antifungal fractions (1 - 3) of *Streptomyces* sp. 9

Figure S7. HPLC chromatogram of purified antifungal fractions (3 - 6) of *Streptomyces* sp. 10

Figure S1. Utilization of substrates by the paper disk method

<table>
<tr><th>Object</th><th>Row</th><th>Plate</th></tr>
<tr><td rowspan="4">A10</td><td>A</td><td rowspan="4">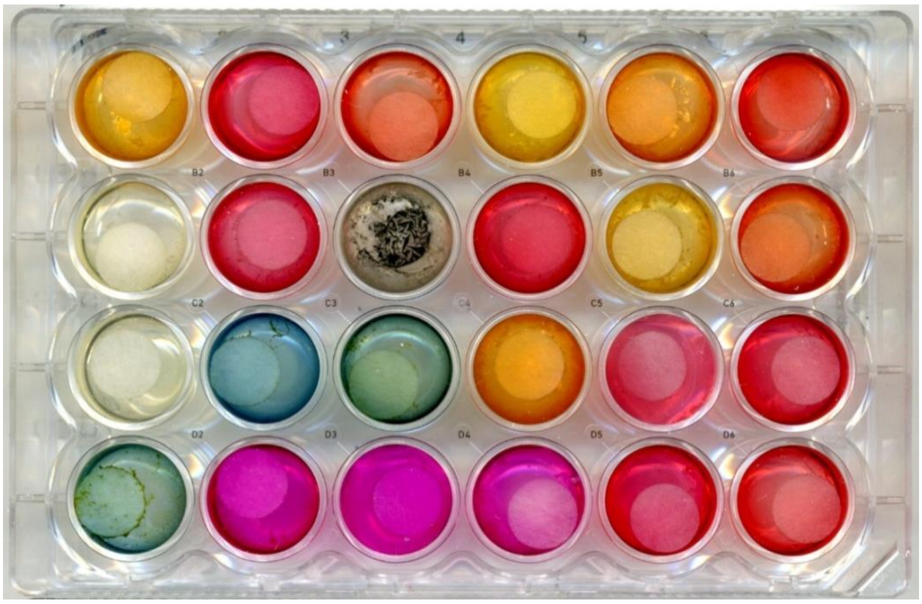</td></tr>
<tr><td>B</td></tr>
<tr><td>C</td></tr>
<tr><td>D</td></tr>
<tr><td rowspan="4">Control</td><td>A</td><td rowspan="4">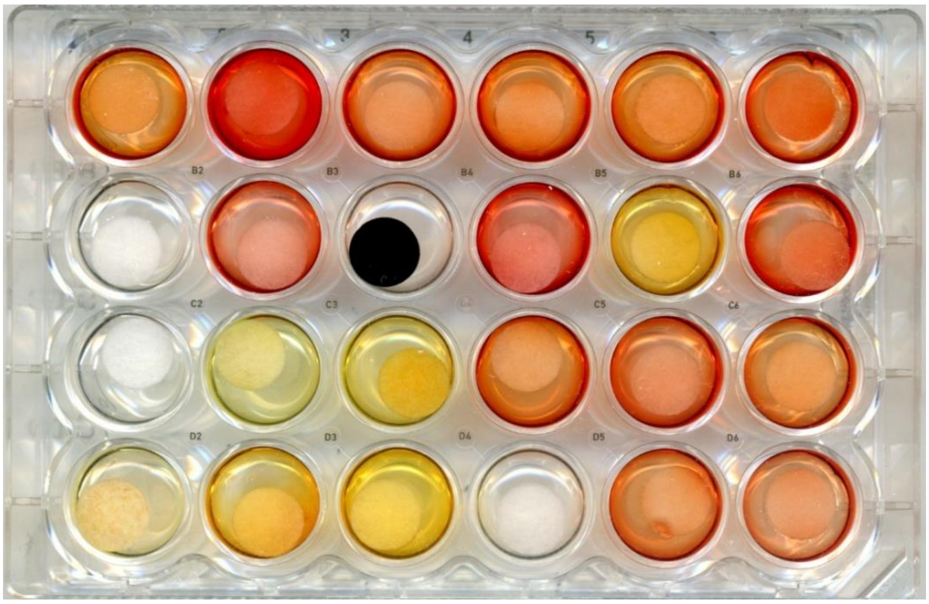</td></tr>
<tr><td>B</td></tr>
<tr><td>C</td></tr>
<tr><td>D</td></tr>
</table>

Row A: 1-mannose, 2- dulcite, 3- adonite, 4 - glucose, 5 - mannitol, 6 – inositol; **Row B**: 1-beta-glucosidase, 2 - salicylate, 3 - gelatin, 4 - lactose, 5 - arabinose, 6 – sucrose; **Row C**: 1 - phenylalanine, 2 - ornithine, 3 - arginine, 4 - maltose, 5 - rhamnose, 6 – sorbitol; **Row D**: 1 - lysine, 2 - citrate, 3 - malonate, 4 - uric, 5 - raffinose, 6 – cellulose.

Back to Contents

Figure S2. Phenotypic properties: utilization as sole carbon source (1.0 %, w/v), growth at NaCl (0.6, 1, 5, 8%) and hydrolysis of urea.

A10 – base

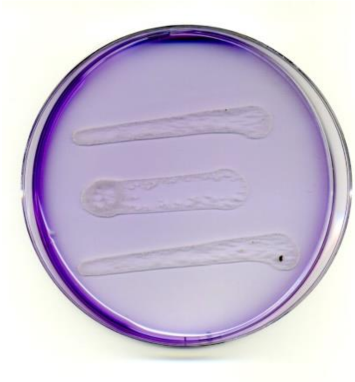

A10 – fructose

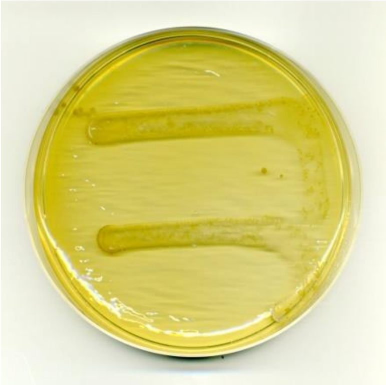

A10 - NaCl-1%

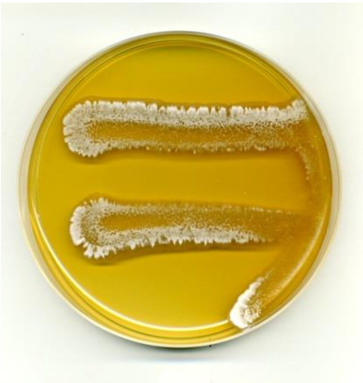

A10 – arabinose

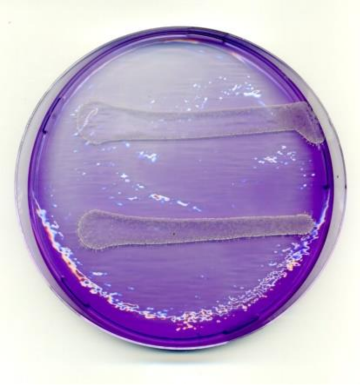

A10 – galactose

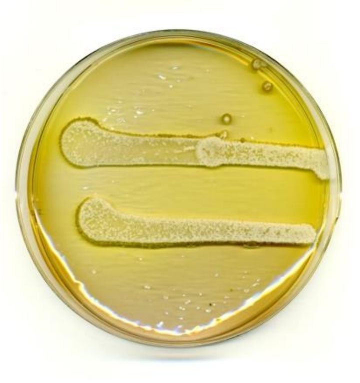

A10 - NaCl-5%

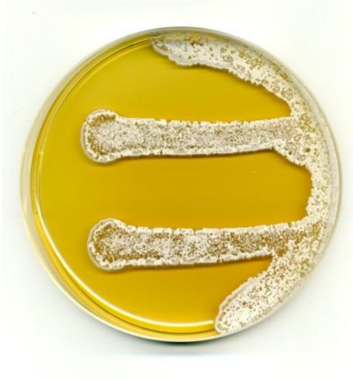

A10 – cellulose

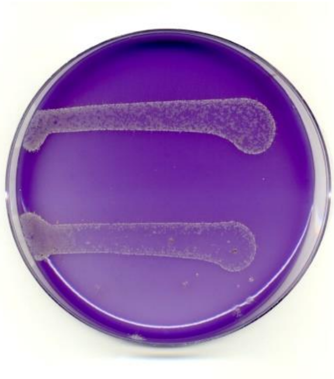

A10 – glucose

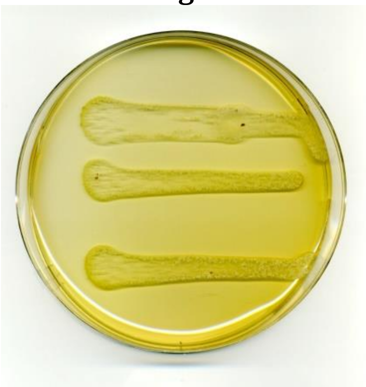

A10 - NaCl-8%

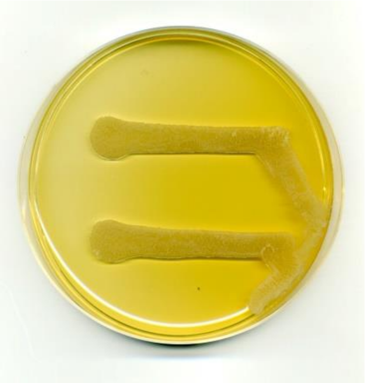

A10 – dextran

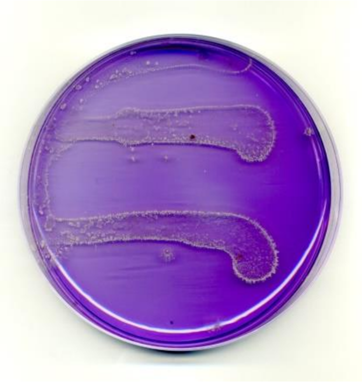

A10 – maltose

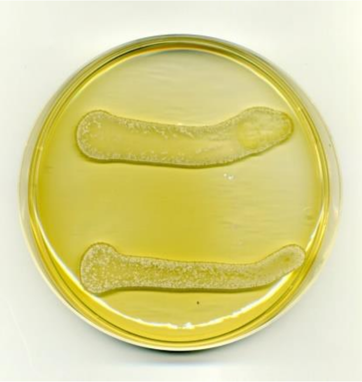

A10 – NaCl-0.6%

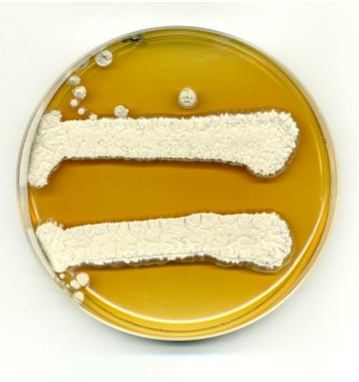

Back to Contents

A10 – inositol

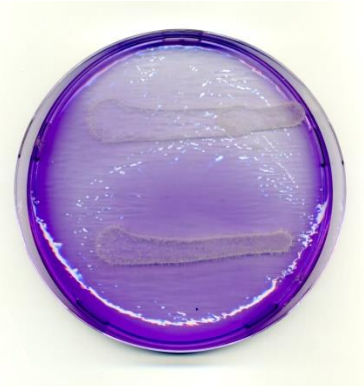

A10 – mannitol

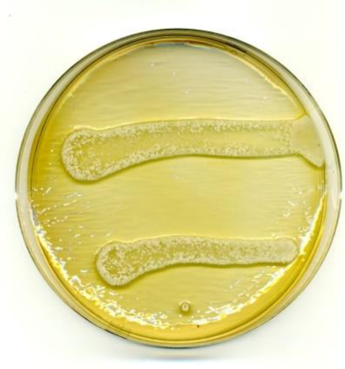

A10 – sucrose

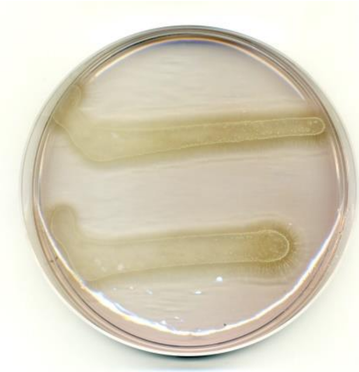

A10 – lactose

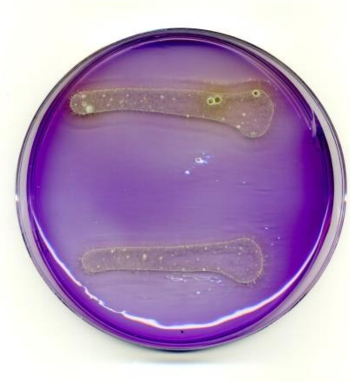

A10 – xylose

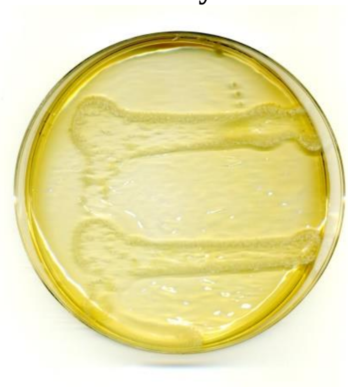

Urease

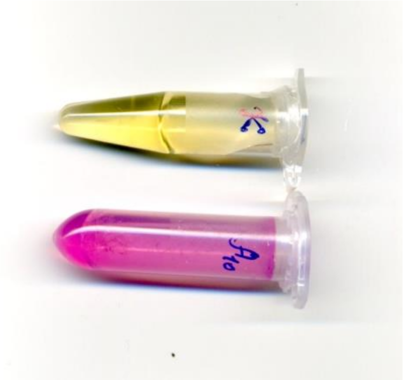

A10 – raffinose

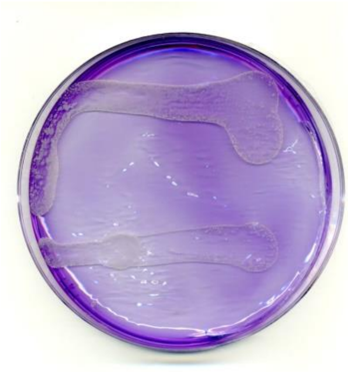

A10 – rhamnose

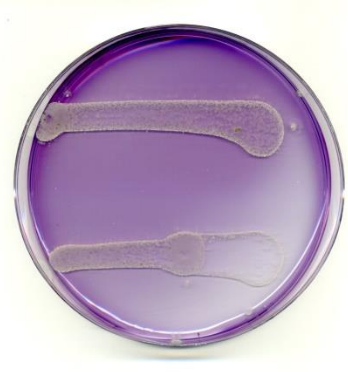

A10 – sorbitol

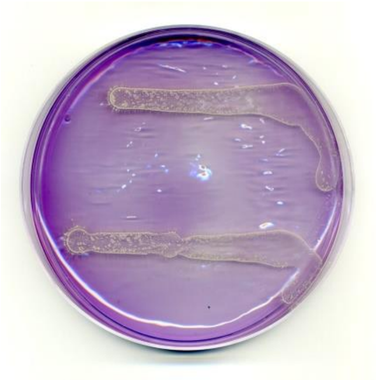

[Back to Contents]

Figure S3. Light microscopy photo of Rectiflexibiles spore chains of strain A10 after cultivation for 2 weeks at 28°C on ISP3 medium (magnification 400)

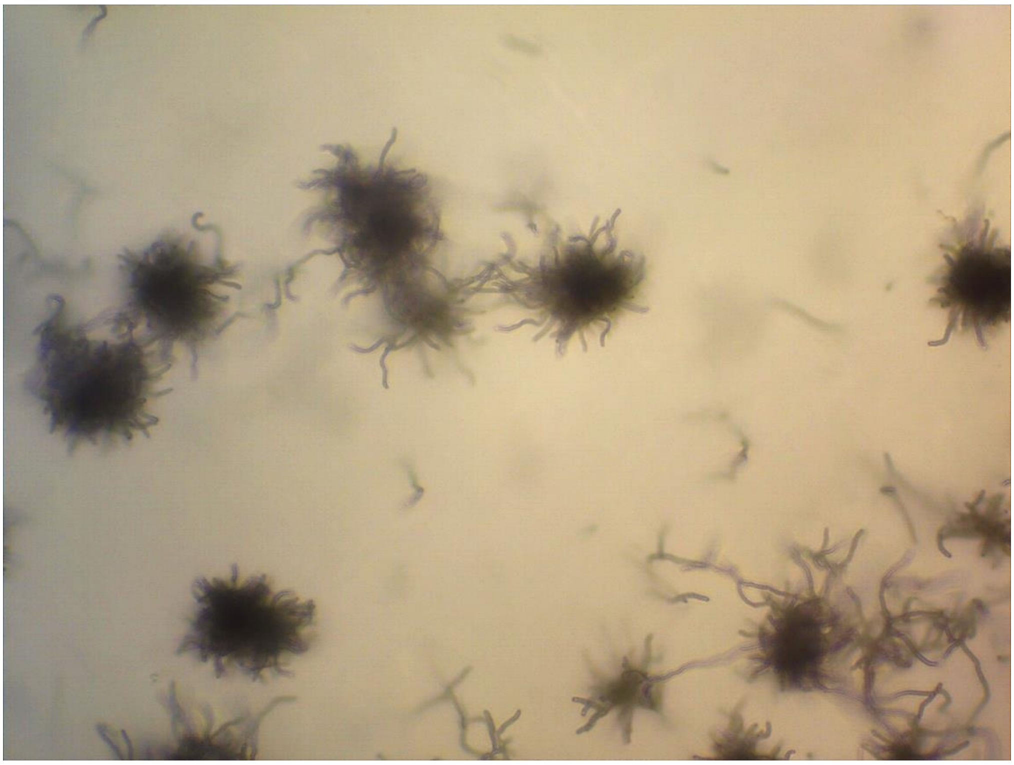

Back to Contents

Figure S4. Antagonistic activity of the *Streptomyces* sp. A10 using dual-culture assay

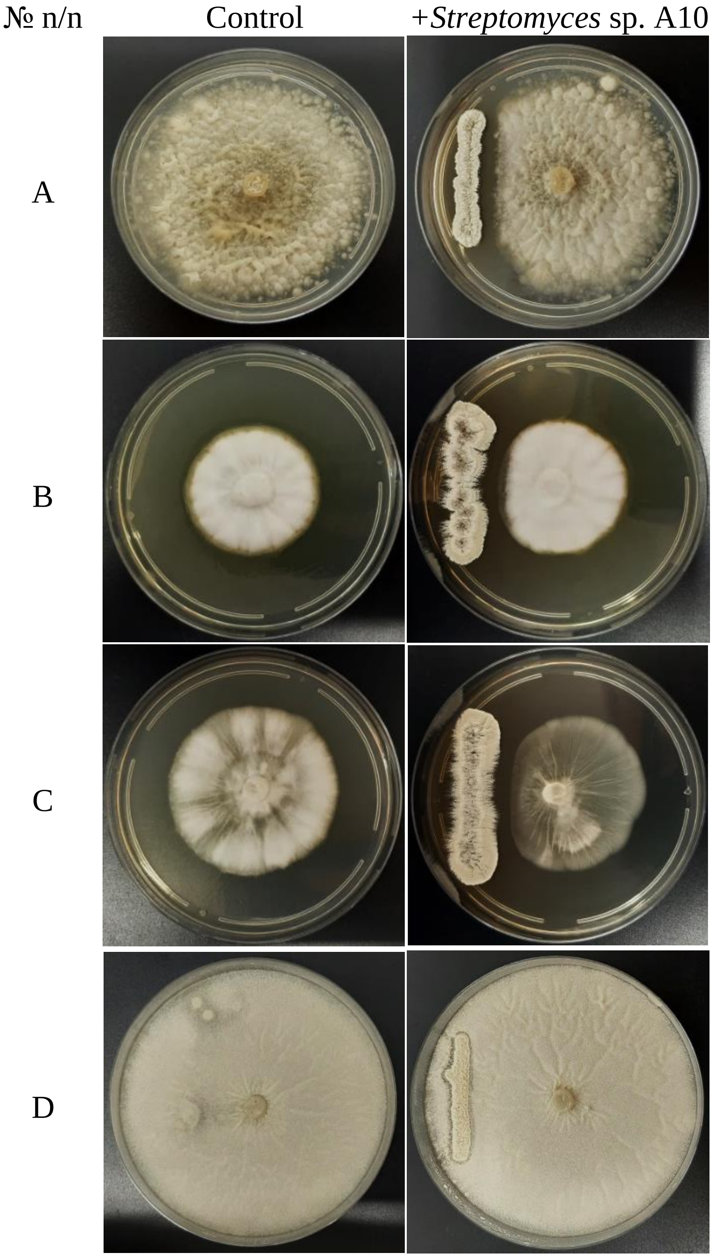

(A) *C. coronatus* VKPM F-442; (B) *O. sinensis* VKPM F-1479; (C) *B. bassiana* VKPM F-1357; (D) *C. coronatus* VKPM F-1359.

Back to Contents

Figure S5. Antagonistic activity of the *Streptomyces* sp. strain A10 using the cross streak method

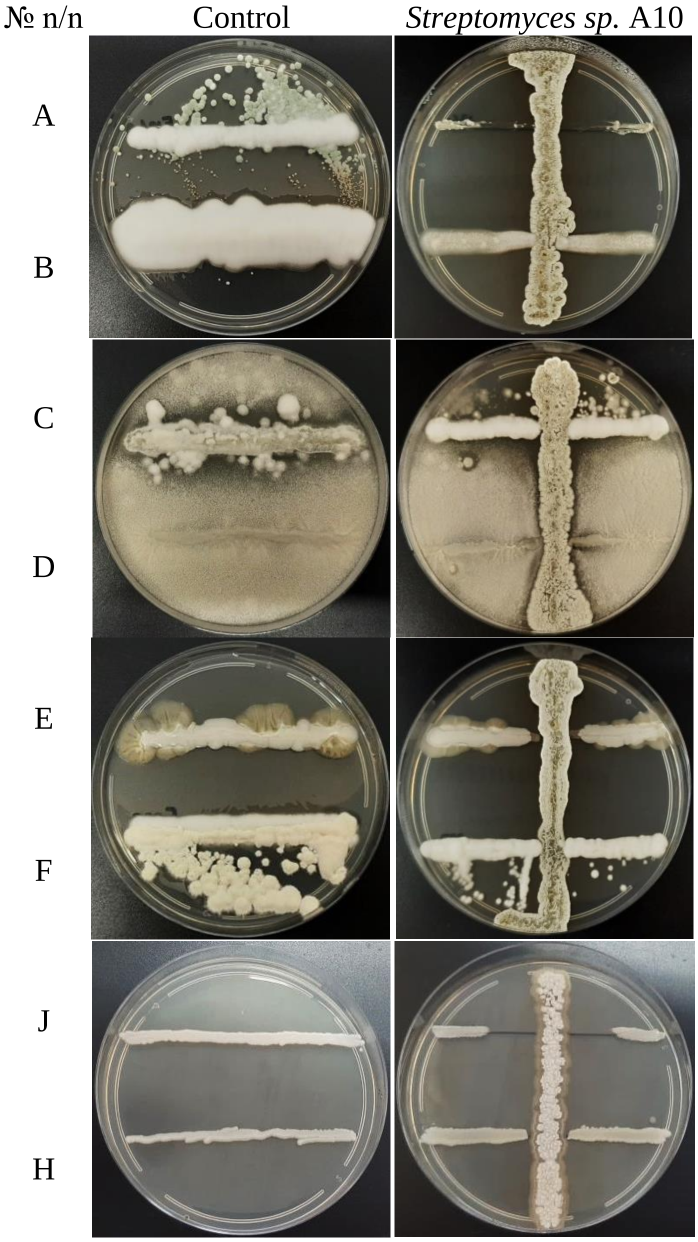

(A) *M. rileyi* VKPM F-1360; (B) *O. sinensis* VKPM F-1479; (C) *B. bassiana* VKPM F-1357; (D) *C. coronatus* VKPM F-1359; (E) *C. coronatus* VKPM F-442; (F) *L. lecanii* VKPM F-837; (J) *C. albicans* CBS 8836; (H) *St. aureus* ATCC 25923.

Back to Contents

Figure S6. HPLC chromatogram of purified antifungal fractions (1 - 3) of *Streptomyces* sp.

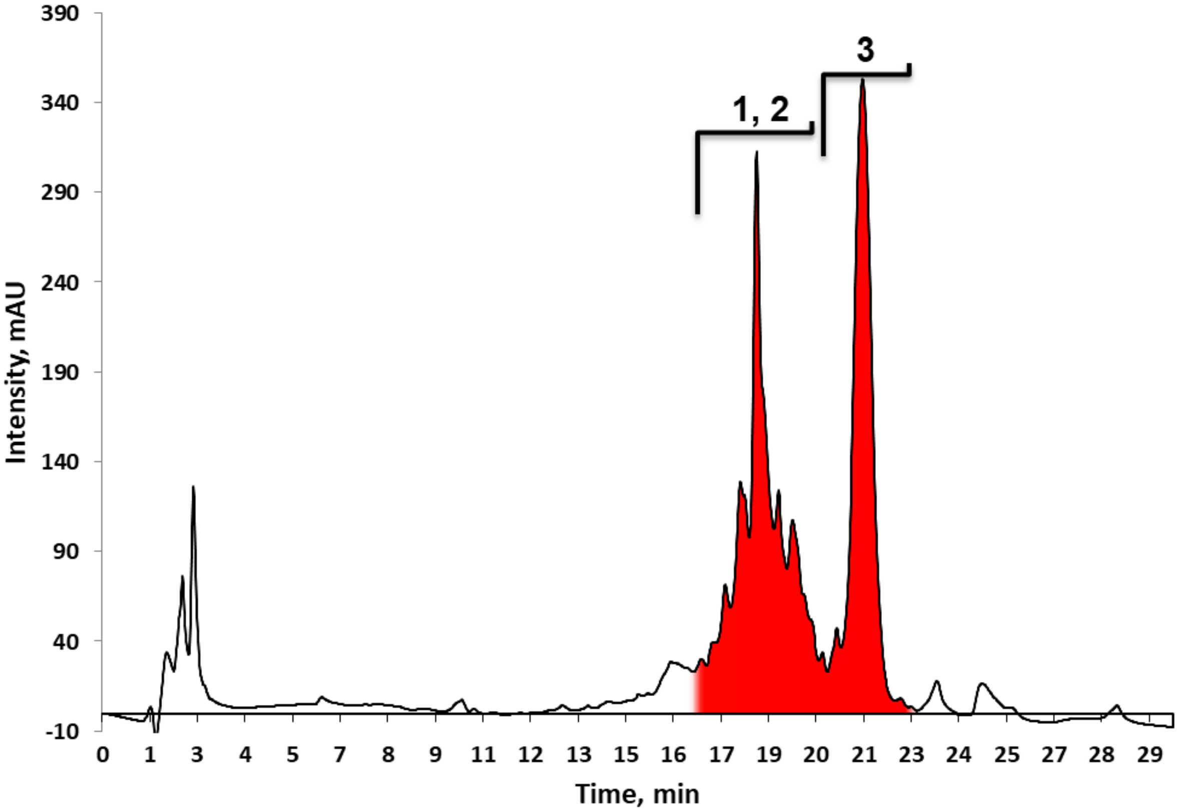

Back to Contents

Figure S7. HPLC chromatogram of purified antifungal fractions (3 - 6) of *Streptomyces* sp.

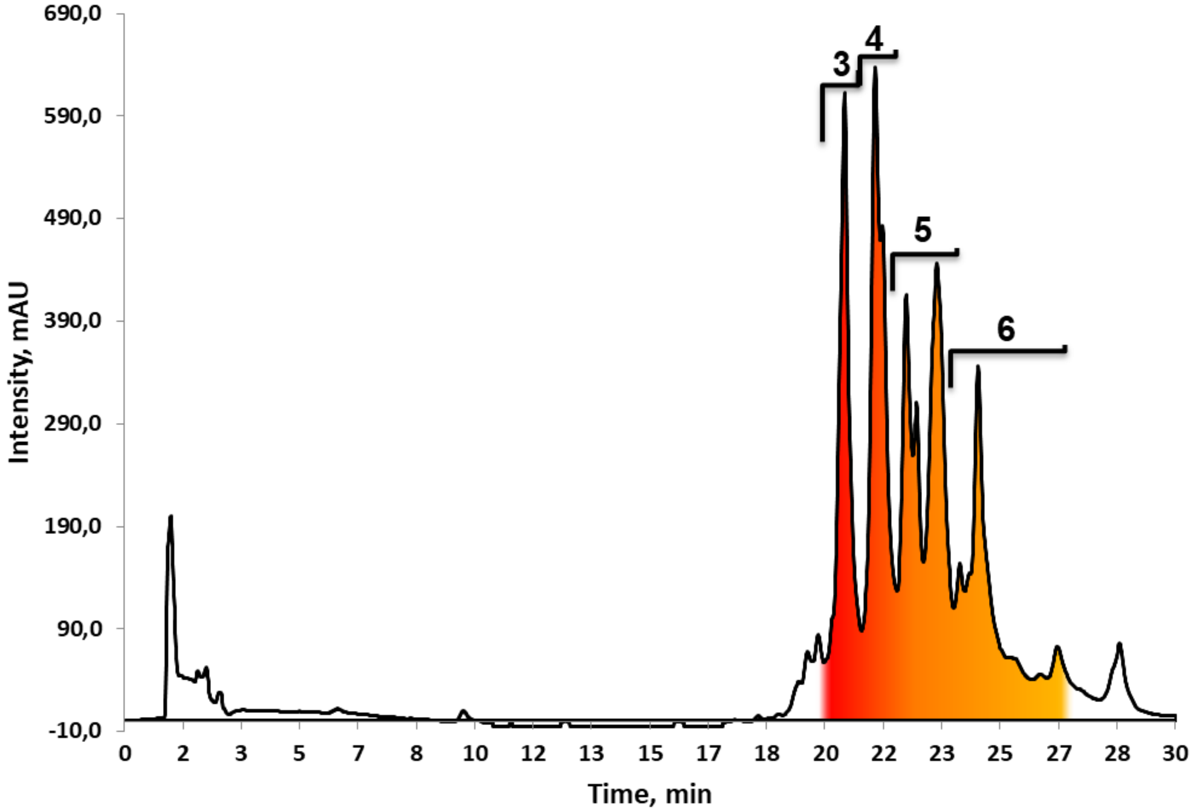

Back to Contents
